# Estimation of ultrasound reference values for the ulnar nerve fascicular number and cross-sectional area in young males: A cross-sectional study

**DOI:** 10.1097/MD.0000000000006204

**Published:** 2017-03-10

**Authors:** Mohamed Abdelmohsen Bedewi, Ahmed M.M. Yousef, Amr Adel Abd-Elghany, Mohamed Sherif el-sharkawy, Ezzat M. Awad

**Affiliations:** a Department of Diagnostic Radiology, College of Medicine, Prince Sattam bin Abdulaziz University, Al-kharj; b College of Applied Medical Sciences, Sattam Bin Abdul-Aziz University; c King Khalid University Hospital, King Saud University, Kingdom of Saudi Arabia; d Department of Vascular Biology and Thrombosis Research, Center for Physiology and Pharmacology, Medical University of Vienna, Vienna, Austria.

**Keywords:** peripheral nerve, reference values, ulnar nerve, ultrasound

## Abstract

The objective of this study is to estimate the reference values for the number of fascicles and cross-sectional area (CSA) of the ulnar nerve at a single predetermined site by ultrasound in healthy young adult males.

The demographic and physical characteristics of 50 adult male volunteers were evaluated and recorded. The subjects were positioned supine with the elbow flexed at 90° and the palm of the hand placed on a hard surface. The ulnar nerve was scanned bilaterally 1 cm proximal to the medial epicondyle in projection of the cubital tunnel. The number of fascicles and mean CSA of the ulnar nerve were identified. In addition, the side-to-side differences of the estimated reference values and their correlations with the age, weight, height, and body mass index (BMI) were evaluated.

The mean fascicular number was 5.66 ± 1.48, the mean ultrasound-estimated CSA of the ulnar nerve was 6.54 ± 1.67 mm^2^ and both sides were comparable in the mean CSA and fascicular number (6.43 ± 1.80 mm^2^ and 5.88 vs 6.64 ± 1.55 mm^2^ and 5.44, for right and left side, respectively). No significant correlations were observed between CSA and fascicles number and age, weight, height, or BMI of study subjects.

The reference values for the number of fascicles number and the CSA of the ulnar nerve at a single predetermined site were identified. These values could be used for the sonographic diagnosis and follow-up of the ulnar nerve lesions.

## 1 Introduction

Recent studies demonstrated an increasing interest in peripheral nerve ultrasonography, which is used for the diagnosis of entrapment neuropathies, posttraumatic nerve evaluation, and other peripheral nerve pathologies.^[1]^ It was widely accepted that the ulnar nerve neuropathy is the 2nd most common type of nerve pathology in the upper extremity, after median nerve neuropathy in carpal tunnel syndrome.^[2]^

Currently, the ulnar nerve, as one of the commonly affected peripheral nerves of the upper limb, can be evaluated with high resolution sonography.^[3]^ The identification of reliable reference values is important in establishing a practical screening test for ultrasonographic evaluation of patients suspected of having peripheral nerve disorders.^[4]^ It is well recognized that the elbow is the commonest site of affliction for ulnar nerve neuropathy, where it passes superficially in the cubital tunnel and become more vulnerable to trauma.^[5]^ An enlarged cross-sectional area (CSA) of the nerve is the key finding in peripheral nerve injuries and is the most widely accepted measurement for diagnosing entrapment neuropathies.^[6,7]^ Previously CSAs of some peripheral nerves have been obtained in the Western^[8,9]^ and Asian populations.^[4,6,10]^ However, there is no such population study that was done for a local community in the middle-east. Moreover, no sufficient data in the literature about the reference values of nerve fascicular number; that could be used in assessment of different types of neuropathies.

## 2 Methods

### 2.1 Participants

The local institutional review board committee approved the study protocol, and all participants provided an informed consent before enrollment. Fifty healthy university male students, 18 to 30 years old, were recruited from December 2014 to May 2015, at a university hospital. For each participant, the age, height, weight, and body mass index (BMI) were recorded before ultrasound scanning. The participants were free from any symptoms of neuropathy especially cubital tunnel syndrome, as indicated by history and clinical examination.

### 2.2 Technique

**Figure 1:**
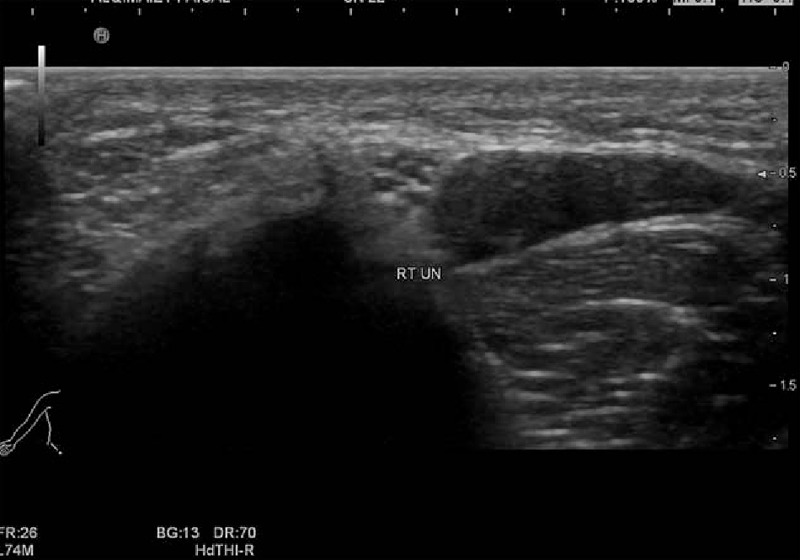
Short axis scan of the ulnar nerve 1 cm before the level of the medial epicondyle cross-sectional area (CSA) with clear fascicular pattern.

The ultrasound scanning of the ulnar nerve was carried out using Hitachi ultrasound diagnostic scanner (Hi vision Avius, Tokyo, Japan) using a 5 to 13 MHZ linear transducer. All studies were performed by the same senior radiologist, experienced in neuromuscular ultrasound. The patients were positioned supine with the elbow flexed at 90° and the palm of the hand placed on a hard surface. The ulnar nerve was scanned bilaterally 1 cm proximal to the medial epicondyle in projection of the cubital tunnel where it was easily identified and measured. The number of fascicles was counted in a short axis view (appearing as rounded and hypoechoic), and the CSA of the ulnar nerve was obtained by scanning in a short axis view, circumferentially tracing inside the hyperechoic rim of the nerve, with the transducer perpendicular to the nerve to obtain accurate values to avoid anisotropy (Fig. 1). Images and results were saved electronically and analyzed.

### 2.3 Statistical analysis

Statistical analysis was performed using Statistical Package for the Social Sciences (SPSS) version 21 software (SPSS Inc., Chicago, IL). All data were presented as mean ± standard deviation (SD) and range. The mean ulnar nerve CSA and fascicles number were compared between both sides using Wilcoxon signed rank test. The correlations between CSA and fascicular number of the ulnar nerve, and age, weight, height, and BMI were evaluated using Pearson correlation coefficient (*r*). A *P* value of <0.05 was considered significant.

## 3 Results

During the recruitment period we studied 50 subjects with a mean age of 21.62 ± 1.70 years (median: 21; range: 18–30). As measured by ultrasound at the level of the medial epicondyle, the mean fascicular number was 5.66 ± 1.48 (median: 6.00; range: 2–9), and the mean ulnar nerve CSA was 6.54 ± 1.67 mm^2^ (median: 6.0; range: 4–10). On the right side, the mean fascicular number was 5.88 ± 1.48 (median: 6.00; range: 2–9), and the mean CSA was 6.43 ± 1.80 mm^2^ (median: 6.00; range 4–11). On the left side, the mean fascicular number was 5.44 ± 1.47 (median: 5.50; range: 2–8), and the mean CSA was 6.64 ± 1.55 mm^2^ (median: 6.50; range 4–10). Both sides were comparable in CSA and fascicular number (*P* = 0.493 and 0.098, respectively). The demographic data, and ulnar nerve CSA and fascicular number of the studied 50 healthy male subjects are summarized in Table 1.

**Table 1:**
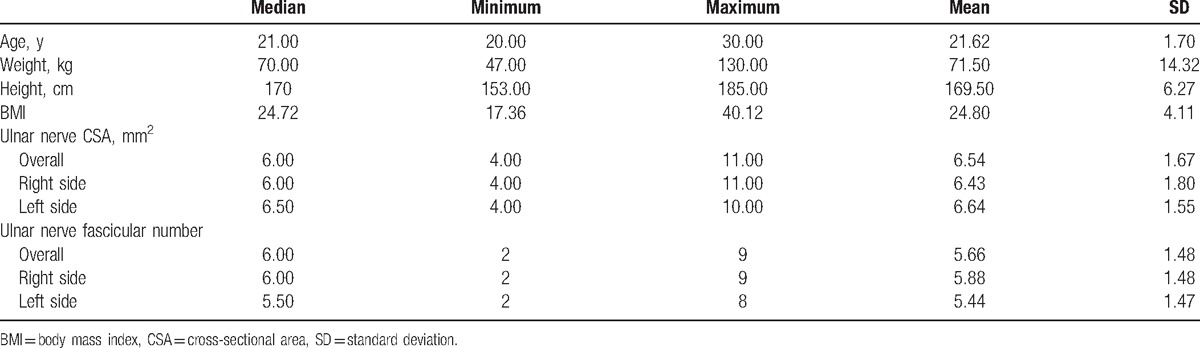
Demographic data, ulnar nerve CSA, and fascicular number of 50 healthy men.

As shown in Table 2, no significant correlation was observed between the number of fascicles of the ulnar nerve and the mean CSA and age, weight, height, or BMI of studied subjects (*P* > 0.05).

**Table 2:**
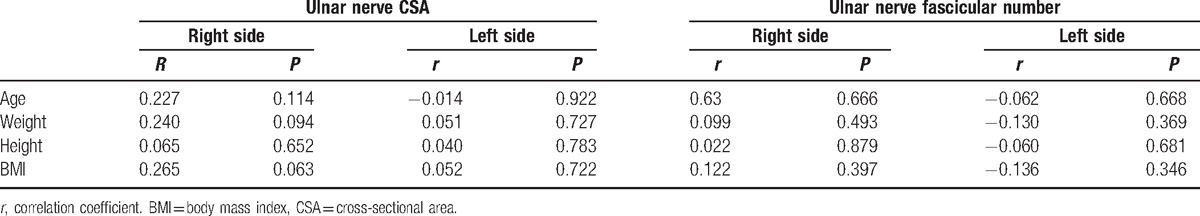
Correlations between age, weight height, and BMI, and ulnar nerve CSA and fascicular number on the right and left sides.

## 4 Discussion

The ulnar nerve has a superficial pathway around the medial epicondyle, where it is most vulnerable to external trauma or compression, particularly at its passage through the cubital tunnel.^[8]^ In the current study, we estimated the fascicular number of the ulnar nerve and the CSA in healthy adult males to obtain standard reference values, which could be important especially in evaluation of posttraumatic nerve injuries. The study included 50 healthy volunteers who represented the same ethnical group to minimize the interracial variations. To the best of our knowledge, this is the first study regarding the reference values for the ulnar nerve number of fascicles.

Although different measurements have been suggested for ulnar nerve ultrasonography, CSA is the most widely accepted and used parameter.^[6]^ Our results showed that the ultrasound-estimated reference value for the ulnar nerve CSA is 6.54 mm^2^. Our finding is nearly similar to that reported by Yalcin et al^[1]^ and Cartwright et al,^[8]^ who measured the ulnar nerve CSA proximal to the medial epicondyle and found that the CSA value is 6.2 and 6.7 mm^2^, respectively. In contrast, Bathala et al^[6]^ studied 100 healthy Asian volunteers to obtain CSAs of normal ulnar nerves at different predetermined sites and reported a CSA value of 4.6 mm^2^, for middle-aged male subjects, at the elbow region. Other investigators reported a higher CSA value of ulnar nerve, Jacob et al^[11]^ reported 7.9 mm^2^.

In accordance with most of previous studies,^[1,2,11]^ no significant difference between right side and left side with respect to CSA and fascicular number of the ulnar nerve. Also, we did not find any significant relationship between the mean CSA of the ulnar nerve and the height of the volunteers. These findings are similar to that previously reported by Bathala et al^[6]^; however, these findings are not consistent with Cartwright et al^[8]^ and Yalcin et al.^[1]^

Despite a positive correlation observed between the ulnar nerve CSA, body weight, and BMI, especially on the right side, this correlation did not reach a significant level (*P* = 0.094 and 0.063). Also, Yalcin et al^[1]^ and Bathala et al^[6]^ observed no correlation between CSA and BMI values. However, Cartwright et al^[8]^ found a positive correlation between CSA and BMI, which depends on the body weight.

Many studies^[6,12]^ found a significant positive correlation between age and CSA of the ulnar nerve. In the present study, no statistically significant relationship between the age of the patient and the CSA of the ulnar nerves was observed. However, the age factor is not applicable in our study as we included only subject with a limited age range.

We reported a reference value of 5.66 for ulnar nerve fascicular number. This value was comparable on right and left sides and had no relationship to age, weight, height, or BMI. The estimation of the reference value of the nerve fascicular number could help in the diagnosis of hereditary sensory and motor neuropathies as well as immune-mediated neuropathies. Despite that, there has been no sufficient research on this issue yet.

### 4.1 Study limitations

The present study has some limitations. The study included only men with limited age range, and the ultrasound-estimation was performed at one reference site. However, the estimation of the reference nerve values for a particular gender and age group at a specific site provide specific information to the clinicians. Future double-arm studies including healthy individuals and patients with ulnar nerve lesions may be warranted to increase our knowledge and to enhance the clinical value of the estimated parameters.

## 5 Conclusion

This is the first study to report fascicular number of the ulnar nerve at a single predetermined site, which could be a primer to be used in the diagnosis and follow-up of the ulnar nerve trauma-related lesions and different types of diffuse and focal neuropathies.
